# Study of needle punctures into soft tissue through audio and force sensing: can audio be a simple alternative for needle guidance?

**DOI:** 10.1007/s11548-021-02479-x

**Published:** 2021-10-15

**Authors:** Muhannad Sabieleish, Katarzyna Heryan, Axel Boese, Christian Hansen, Michael Friebe, Alfredo Illanes

**Affiliations:** 1 INKA-Innovation Laboratory for Image Guided Therapy, Otto-von-Guericke University Magdeburg, Magdeburg, Germany; 2 Department of Measurement and Electronics, AGH University of Science and Technology, Kraków, Poland; 3 Research Campus STIMULATE, Sandtorstrasse 23, 39106 Magdeburg, Germany

**Keywords:** Audio guidance, Force feedback, Needle interventions, Needle puncture

## Abstract

**Purpose:**

Percutaneous needle insertion is one of the most common minimally invasive procedures. The clinician’s experience and medical imaging support are essential to the procedure’s safety. However, imaging comes with inaccuracies due to artifacts, and therefore sensor-based solutions were proposed to improve accuracy. However, sensors are usually embedded in the needle tip, leading to design limitations. A novel concept was proposed for capturing tip–tissue interaction information through audio sensing, showing promising results for needle guidance. This work demonstrates that this audio approach can provide important puncture information by comparing audio and force signal dynamics during insertion.

**Methods:**

An experimental setup for inserting a needle into soft tissue was prepared. Audio and force signals were synchronously recorded at four different insertion velocities, and a dataset of 200 recordings was acquired. Indicators related to different aspects of the force and audio were compared through signal-to-signal and event-to-event correlation analysis.

**Results:**

High signal-to-signal correlations between force and audio indicators regardless of the insertion velocity were obtained. The force curvature indicator obtained the best correlation performances to audio with more than $70\%$ of the correlations higher than 0.6. The event-to-event correlation analysis shows that a puncture event in the force is generally identifiable in audio and that their intensities firmly related.

**Conclusions:**

Audio contains valuable information for monitoring needle tip/tissue interaction. Significant dynamics obtained from a well-known sensor as force can also be extracted from audio, regardless of insertion velocities.

## Introduction

Percutaneous needle insertion into soft tissues is a prominent part of minimally invasive procedures (MIPs). It is performed for several applications such as needle biopsies, Veress needle insertion during laparoscopic access, spine- and neurosurgery, renal access, brachytherapy, and regional anesthesia [2, 6, 18].

In these procedures, surgeons must insert the needle to reach a target area. The precision in the execution of this procedure is of the most importance. On the one hand, e.g., in biopsy procedures, the exact location of the tissue collection has to be ensured to avoid false-negative results, and on the other hand, a collision of the needle with adjacent structures has to be prevented as it leads to high risk of damage and bleeding-related complications. For this reason, the level of the surgeon’s professional expertise plays a vital role in the success of such procedures. Usually, less experienced surgeons have more difficulties assessing the needle tip’s interactions with soft tissue, given the reduced tactile sensation transmitted via the instruments. Hence, to prevent complications and overcome the long learning curve, there is an immense need to provide the surgeon with accurate information on percutaneous needle tip localization and haptic feedback.

Nowadays, different imaging technologies, such as ultrasound (US), computed tomography (CT), and magnetic resonance imaging (MRI), are used to support clinicians. However, they face limitations due to image artifacts such as bevel line artifacts in US-guidance [20], metal artifacts in CT-guidance [15], and susceptibility artifacts in MRI-guidance [23]. Therefore, optical or electromagnetic tracking devices have also been used, but they imitate the range of freehand maneuvers, and the external hardware for tracking imposes an extra cost on the procedure and makes it cumbersome.

Sensor-based solutions have been proposed to provide additional guidance haptic feedback during MIPs. These needle-embedded sensing techniques are based mainly on measuring force through strain gauge [4] and fiber Bragg grating sensors [1, 5, 7, 12, 14] or measuring impedance [13, 17]. Although they could provide significant information concerning puncture events, they require the sensors to be embedded in the needle tip or shaft in direct contact with the patient’s organs and tissue, leading to sensor integration difficulties, loss of tool functionality due to required cabling, sterilization issues, and complex certification procedures. These challenges limit these solutions from being used in real clinical environments [4, 13, 18].

Proximally located force sensors have been widely used for the analysis of percutaneous needle insertion into soft tissue [2, 6, 16, 24, 25]. However, they have been mainly used for better understanding the mechanical characteristics of the needle tip and tissue interactions and not for real clinical application. Although they can be considered a reference for evaluating other sensing techniques, their slow dynamical response, high cost, and high sensitivity to user make these sensing techniques challenging to be adopted for regular clinical use.

In order to overcome these drawbacks, an audio-based technique has recently been introduced in [11]. This concept consists of *listening* to the instrument’s tip–tissue interactions with an audio sensor located at the proximal end of the instrument. The audio signal resulting from these interactions starts at the instrument’s tip and then naturally propagates through its shaft, without any active element. The generated signal can then be processed to extract useful guidance information that could be mapped into feedback to surgeons. The main advantages of this approach are that no sensor is needed to be placed in direct contact with the patient organs and tissues and that it is possible to listen to standard tools with no alteration of their functionality.

This novel method showed promising results for needle guidance applications such as an indication of tissue passage [10] or for characterizing and classifying a needle perforation/puncture [9, 10, 19, 22].

The main purpose of this work is to demonstrate that audio can contain valuable dynamical information concerning interactions between the needle tip and the tissue. For this purpose, we compare the acoustic dynamics of the needle insertion process provided by audio guidance with the information acquired by the force sensor. The main idea is to relate events and dynamical characteristics extracted from the audio signal with those extracted from the force signal through indicators and event features computed by processing both signals. This work does not intend to replace a force sensor but to prove that from audio we can derive the information about important events that are also present in the force signal during needle insertion. Force is used for comparison purposes because force measures are a reliable reference for needle punctures, as shown in the literature [2, 6].

For this purpose, audio and force signals were first synchronously recorded. The audio signal was processed by extracting its homomorphic envelope. Indicators related to local event intensity, derivative, and curvature were computed from the force signal. The Pearson coefficient was used for signal-to-signal correlation between audio and force indicators. An event-to-event analysis correlation between audio and force events was then performed by computing features from the indicators.

**Fig. 1:**
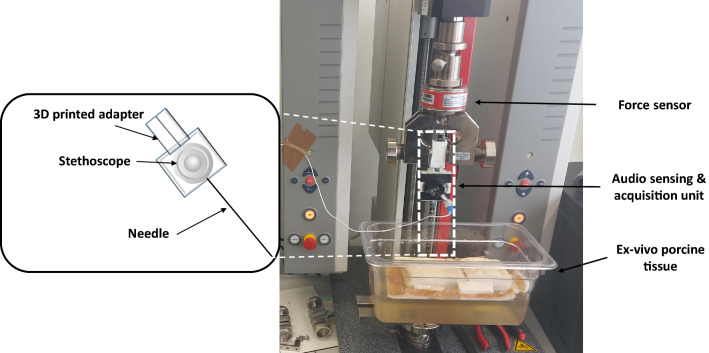
Experimental setup for data acquisition of needle insertions into an ex-vivo porcine phantom

Preliminary results of this work have been presented in [9], where 80 needle insertion recordings were performed at an insertion velocity of 3 mm/s, showing high signal-to-signal and event-to-event correlations. However, the comparison was made by performing experiments with only one insertion velocity. The main contribution of this new work is to demonstrate that the dynamical relations between audio and force are independent of the velocity with which the needle is inserted and that the previously observed dependencies can be generalized. The experiment performed in [9] was repeated with three additional insertion velocities, and a slightly different signal processing algorithm than [9] was used for computing the audio and force indicators.

Results show that audio and force characteristics, regardless of the insertion velocity, are firmly related, although both sensors have entirely different specifications and properties. This work affirms the results presented in [9]: audio solely can contain valuable information for monitoring needle tip/tissue interaction dynamics since significant dynamics that can be obtained from a well-known sensor as force can also be extracted from the audio itself. Moreover, the results show that the audio guidance approach is robust to insertion velocities.

## Experimental setup and data acquisition

The same experimental setup used in [9] is employed in this work (Fig. 1) to acquire audio and force datasets at different insertion velocities. For the audio acquisition, a stethoscope connected to a microphone attached to the needle’s proximal part (18G, 200 mm length biopsy needle—ITP, Germany) via a 3D printed adapter was used. The needle was fixed to a testing machine (Zwicki, Zwick GmbH & Co. KG, Ulm), which was used to perform the automatic insertions and to acquire the axial needle insertion force data. The insertions were performed into an ex-vivo porcine tissue placed on a gelatine layer. The audio and force sampling frequencies were equal to 44100 Hz and 100 Hz, respectively.

Additionally to the 80 recordings performed at 3 [mm/s] from the dataset acquired in [9], three new datasets were acquired with three different velocities of needle insertion, 6, 10, and 14 [mm/s].

A dataset of 40 recordings per insertion velocity was acquired. Table 1 shows a summary of the dataset characteristics.

**Table 1:** Summary of the acquisition of force and audio signal at different needle insertion velocities

Insertion Vel.	Nb. of recordings acquired	$F_s$ audio	$F_s$ force
3 mm/sec.	80		
6 mm/sec.	40		
10 mm/sec.	40	44100 Hz	100 Hz
14 mm/sec.	40		

### Data preparation

**Fig. 2:**
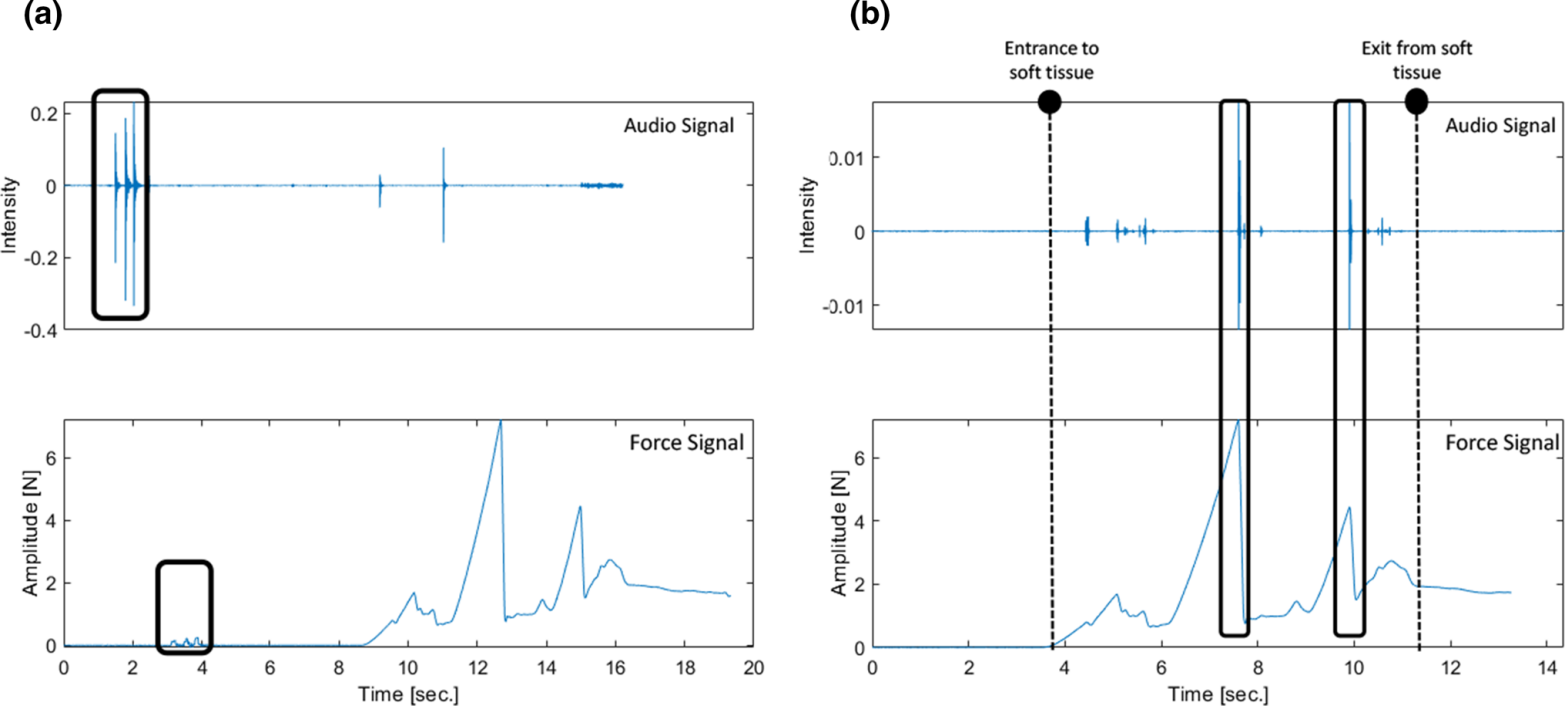
**a** Unsynchronized force and audio data highlighting the triggers used for synchronization, **b** synchronized audio and force signals where the needle’s entry and exit time instants and two main puncture events can be visualized

At the beginning of each recording, triggers were generated by tapping the needle three times. Force and audio signals were synchronized using this trigger event visible in both signals. An example of these signals recorded during the same insertion before and after the synchronization is presented in Fig. 2. It is possible to see in Fig. 2b that the two main punctures during the insertion are correctly synchronized.

Signal segments from audio and force signals were extracted, taking as references the time instants corresponding to 0.5 [s] after the last trigger event and 1 [s] after the tissue exit.

## Audio and force signal processing strategy

In percutaneous procedures, the needle is inserted toward the region of interest (ROI) by needle puncturing different tissue layers such as skin, muscles, and fatty tissue. Puncturing requires different amounts of force depending on the tissue type. A puncture identification could provide meaningful complementary information to image-based needle guidance [14, 25].

**Fig. 3:**
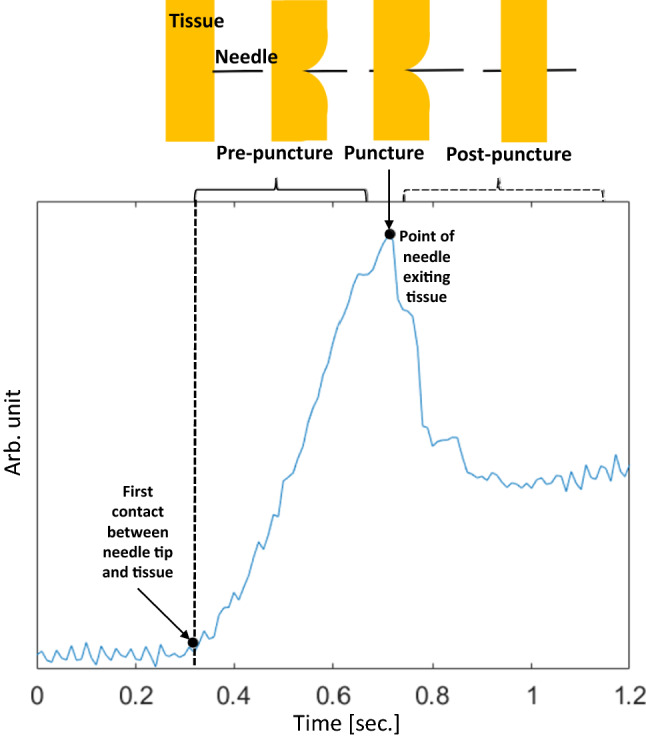
Needle insertion phases into soft tissue according to the force signal

According to the literature [2, 3, 6, 16], three subsequent phases can be distinguished in force during needle puncture (see Fig. 3). Phase 1, a pre-puncture phase, starts when the needle touches the tissue border, resulting in a steadily arising force curve. As soon as tissue breakage starts and a crack is initiated, phase 2 begins. The accumulated energy is released and a sudden drop in force can be observed. Then, in phase 3 (post-puncture), the needle passes through the tissue with continuous friction between the needle shaft and the tissue. This phase might have multiple deflections and cutting phases, depending on the tissue’s type and thickness.

**Fig. 4:**
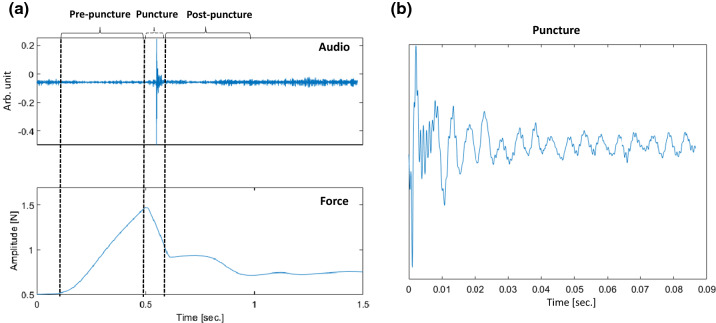
**a** Needle insertion phases into single layer material, **b** zoomed puncture segment audio excitation indicating its transient characteristic

Figure 4a displays a recorded audio event and its corresponding force of an exemplary ideal needle puncture acquired using a simple phantom involving a thin plastic layer immersed in gelatin at 3 cm depth. The insertion was performed following the same setup presented in Sect. 2. It is possible to observe that no audio excitation occurs during the first phase (pre-puncture) since no tissue breakage or structure collision occurs. The second phase’s puncture produces a significant audio excitation due to the audio generated at the tip–tissue breakage point. The post-puncture of phase 3 appears in the audio as a damped oscillatory response mainly due to the excited state of the needle tip and the friction between its shaft and the tissue (Fig. 4b). It can be noticed in the figure that there is a small delay between force and audio signals. This delay happens because of tiny synchronization errors that can occur with the trigger event approach explained in Fig. 2.

**Fig. 5:**
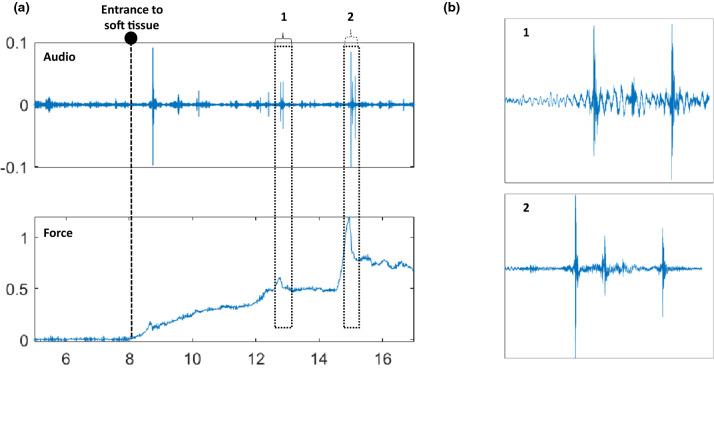
**a** Audio and force signals during needle insertion into ex-vivo tissue, indicating two main events in the force that are identifiable in the audio, **b** zoomed events in the audio signal

Figure 5 shows the audio and force signals of the dataset generated in Sect. 2, during insertion of the needle in real ex-vivo tissue. It is possible to see that the puncture during the second phase and following collisions, friction, and new punctures of inner tissues, can produce complex audio excitation dynamics. In Fig. 5a, two main puncture events identifiable in the force signal are displayed together with the resulting audio excitation. When each audio response is zoomed (Fig. 5b), we can see that one single puncture event results in a complex succession of audio excitations. This is due to the tissue’s inhomogeneity characteristics resulting in multiple interactions and tiny transitions between the needle tip and the tissue. However, even if an audio response is complicated, its dynamics should be related to the dynamical characteristics of the force during the second and third phases. This is what we want to explore in this work.

**Fig. 6:**
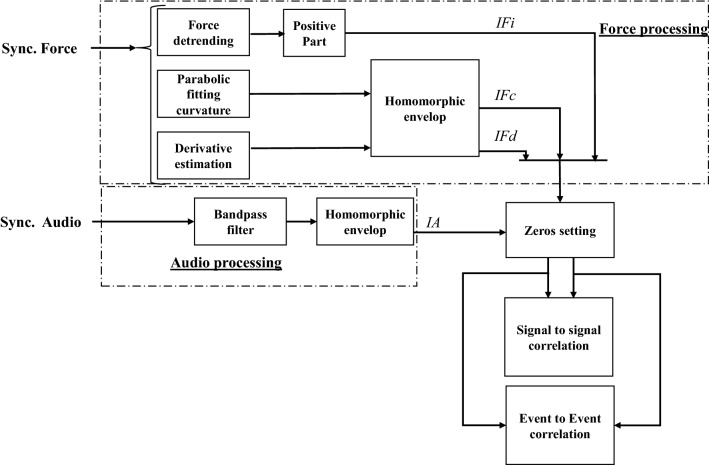
General block diagram with the strategy for audio and force signal processing and for correlating both signals

We aim to extract feature indicators from the force related to the audio excitation’s dynamical characteristics. Three types of features are extracted from the force: local intensity, derivative, and curvature. The local intensity of the detrended force aims to emphasize the increase in force from a local deflection (contact of needle tip with the tissue) passing through its peak (puncture event) and coming back to a steady stage. The derivative features aim to enhance the cumulative energy stored during the boundary displacement, and the curvature to emphasize the fast drop in force just after the puncture. We study the relationship of these force features with an audio feature that is extracted using the homomorphic envelope.

### Audio and force signal processing strategy

Figure 6 displays a block diagram with the main steps to relate audio and force features. First, the audio signal and the force signal are processed in order to compute the different indicators extracted for enhancing the signal features that want to be compared: one audio indicator (*IA*), and three force indicators, related to the local intensity ($IF_{i}$), to the curvature ($IF_{c}$), and the derivative ($IF_{d}$). A signal-to-signal correlation is then performed between audio and force indicators to assess similarity between features and optimize the parameters of the processing algorithms. Finally, an event-to-event correlation is performed by identifying puncture events and assessing the intensity in both audio and force indicators.

It is important to mention that this is a comparative analysis and the real-time requirement is not necessary. All the algorithms in this section were implemented offline.

#### Audio indicator computation

The audio signals were first pre-processed using a 7th-order Butterworth bandpass filter (3000–6000 Hz) to enhance the tissue breakage related information [10]. As already shown in Fig. 5, the set of events involved in one audio excitation denotes the accumulation of energy during the puncture event. This accumulation of energy can be emphasized using a homomorphic envelope [21] over the filtered signal, representing the signal’s amplitude modulation and the energy event accumulation. The computed homomorphic envelope is considered as the audio indicator (*IA*).

**Fig. 7:**
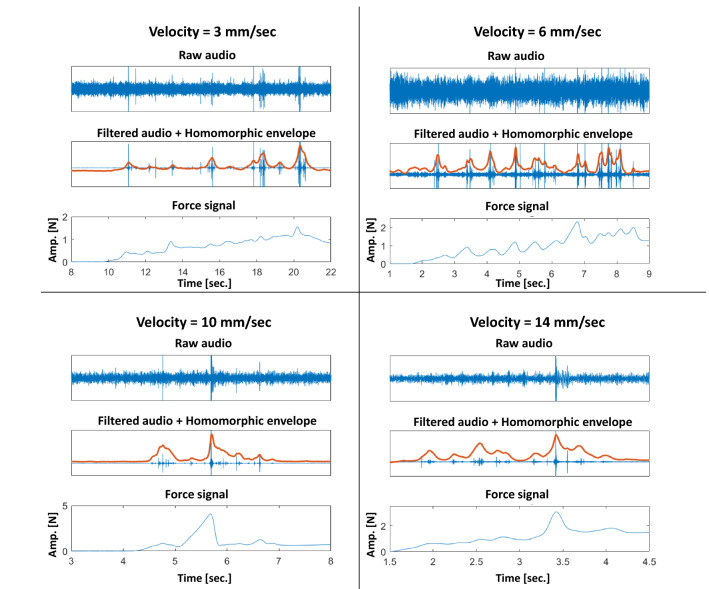
Examples of obtained audio homomorphic envelopes and comparison with force for different insertion velocities

Figure 7 displays four examples of audio processing performed in recordings belonging to the implemented dataset, one example per velocity. It is possible to observe how the bandpass filter enhances the set of audio excitations and how the homomorphic envelope can simplify the dynamics of an excitation, allowing to better visualize the similar audio and force responses.

#### Force indicators computation

Three indicators are extracted from the force: the intensity indicator ($IF_i$), the curvature indicator ($IF_c$), and the derivative indicator ($IF_d$).

$IF_i$ intends to extract the information related to the local intensity of the force. In this aim, the force signal was first detrended by subtracting to signal its baseline. The baseline estimation method was changed compared to the one proposed in [9] to better take into account the force deflection before and after a puncture. For this purpose, the valleys preceding and following a force puncture peak were first detected, and then a linear interpolation was performed between all the detected valleys. Finally, ($IF_i$) was computed as the positive part of the resulting detrended force signal.

The curvature and the derivative force indicators are computed with the same method presented in [9]. For the $IF_c$ computation, the force signal was first approximated using a 2nd degree polynomial within a symmetric sliding window to emphasize and estimate the curvature coefficient. $IF_c$ corresponds to the homomorphic envelope of the estimated curvature.

For the $IF_d$ computation, a derivative filter followed by a smoothing first-order low-pass filter is applied to the force signal. $IF_d$ is computed by extracting the homomorphic envelope of the estimated derivative.

**Fig. 8:**
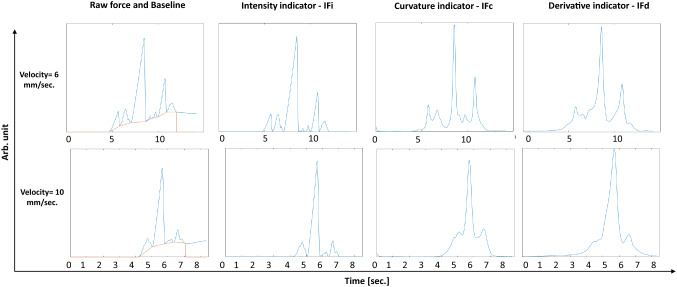
Examples of computation of force baseline and the three proposed force indicators for two different needle insertion velocities

Figure 8 shows results for the new force detrending method and examples of the three extracted indicators with insertions at two different velocities, 6 and 10 [mm/s].

**Fig. 9:**
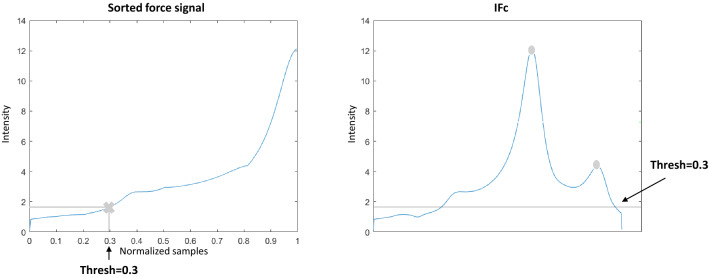
**a** Intensity threshold computation for force events detection, and **b** resulting threshold in the force indicator

#### Force and audio signals correlation methodology

In this work, the relationship between the computed audio and force indicators was evaluated using signal-to-signal and event-to-event correlation methods. As shown in the block diagram of Fig. 6, before correlation analysis, the signal segments of force and audio indicators that do not involve needle penetration (before tissue entry and after tissue exit) are set to zero, as our main interest is the signal segment related to needle tip–tissue interaction.

As we could see in Sect. 3 the computation of the audio and force indicators requires setting some parameters, and their optimization was performed from the signal-to-signal correlation and then used for the event-to-event correlation. The parameters to optimize are the following:

- The cut-off frequency of the low-pass filter used to compute the homomorphic envelope for the audio and force indicators, denoted as $lpf_a$ and $lpf_f$, respectively.
- $h_{win}$ denoting half of the length of the symmetric sliding window around the central signal sample, used for the computation of $IF_c$.

These parameters were optimized to obtain the highest average Pearson correlation coefficient among each insertion velocity dataset and between each pair of audio and force indicators independently (*IA* vs $IF_i$, *IA* vs $IF_d$, *IA* vs $IF_c$). For each set of parameters, the Pearson coefficient is computed per recording and the average Pearson coefficient of the set of signals is then calculated. One parameter is first tuned, and the maximal average coefficient is subsequently identified. Afterward, the other parameters are separately tuned and fixed on a new maximal average Pearson coefficient. Parameters tuning is performed using an exhaustive search optimization scheme within a defined range.

**Fig. 10:**
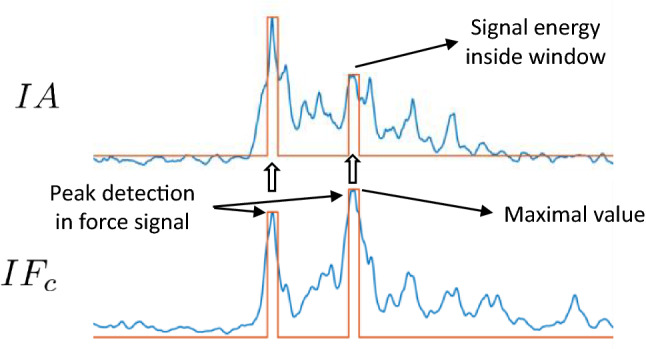
Puncture events detection in $IF_c$ and corresponding window in *IA* for audio excitation analysis

**Table 2:** Average optimized Pearson coefficients for the signal-to-signal correlation between the three force indicators and the audio indicator for the datasets recorded at different velocities

Insertion velocity	IA versus IF	Audio Par.	Force Par.		Mean ± std.	Opt. $\rho$
		$lpf_a$	$h_{win}$	$lpf_f$		
3 mm/sec	*IA* versus $IF_i$	1	n/a	17	0.4375 ± 0.0374	0.5114
	*IA* versus $IF_c$	1	7	1	0.5020 ± 0.0592	0.6482
	*IA* versus $IF_d$	1	n/a	1	0.4721 ± 0.0196	0.5711
6 mm/sec	*IA* versus $IF_i$	1	n/a	19	0.4081 ± 0.0648	0.5627
	*IA* versus $IF_c$	1	1	1	0.6340 ± 0.0456	0.7783
	*IA* versus $IF_d$	1	n/a	1	0.4396 ± 0.0923	0.6888
10 mm/sec	*IA* versus $IF_i$	1	n/a	1	0.4831 ± 0.0462	0.6188
	*IA* versus $IF_c$	1	1	1	0.6270 ± 0.0352	0.7530
	*IA* versus $IF_d$	1	n/a	1	0.5031 ± 0.0510	0.6445
14 mm/sec	*IA* versus $IF_i$	1	n/a	1	0.6063 ± 0.0571	0.8045
	*IA* versus $IF_c$	1	1	1	0.6911 ± 0.0377	0.8362
	*IA* versus $IF_d$	1	n/a	1	0.5882 ± 0.0495	0.8060

For the event-to-event correlation, significant puncture events, identifiable by a significant peak in the force indicator signal, were detected using a standard peak detector algorithm followed by a thresholding step. Then, from the detected force event time instant, a time-domain window is generated in the audio signal to analyze the audio excitation that corresponds to the force detected event.

The threshold was adapted to each force signal indicator by first sorting the indicator in ascending order and then selecting the indicator value located at $30\%$ of the sorted signal’s length (see Fig. 9a).

In Fig. 10 an example of event detection in $IF_c$ and the corresponding audio indicator signal intensity inside the window is shown. Two significant events were detected in the force, and then two windows are generated for analyzing the audio excitation.

Two types of event-to-event dependency analyses were performed. The first one concerns the comparison of maxima detected values within related events in the audio and force indicators through a scatter plot visualization. The second one allows for a quantitative assessment of the relationship between puncture events detected in the force signal with a corresponding identifiable event in the audio signal in the form of a confusion matrix. The main idea is to confirm if the presence of an important puncture event in the force signal also occurs in the audio signal.

## Results

### Signal-to-signal correlation

Table 2 presents the results of the signal-to-signal correlation between audio and force obtained for the four insertion velocities and per each compared pair of audio and force indicator. It is possible to see the optimized parameter values for the extracted indicators ($lpf_a$, $lpf_f$, and $h_{win}$) and the Pearson coefficient’s mean and standard deviation obtained during the optimization procedure. The table shows in its last column the average optimal Pearson coefficient value (Opt. $\rho$) computed from the full set of signals. The optimized Pearson coefficients for all the velocities and force indicators show values over 0.5, which is high considering the different nature of the type of sensors we are comparing.

Also, it is important to notice that for all the velocities, the best results were always obtained for $IF_c$, and the lowest results for $IF_i$. These results suggest that the audio excitation dynamics are rather managed by the elasticity of the tissue punctured than by the force that is required to produce this puncture.

Another important observation is that the best Pearson coefficients were obtained for the highest insertion velocity of 14 [mm/sec]. This might be related to the fact that when the velocity increases, the audio SNR also increased.

**Fig. 11:**
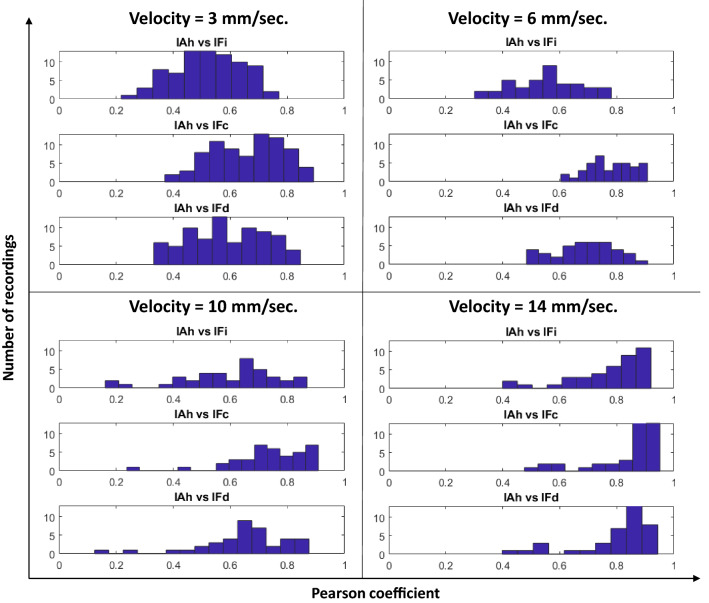
Histograms of Pearson coefficients for all the indicators and for every insertion velocity

**Fig. 12:**
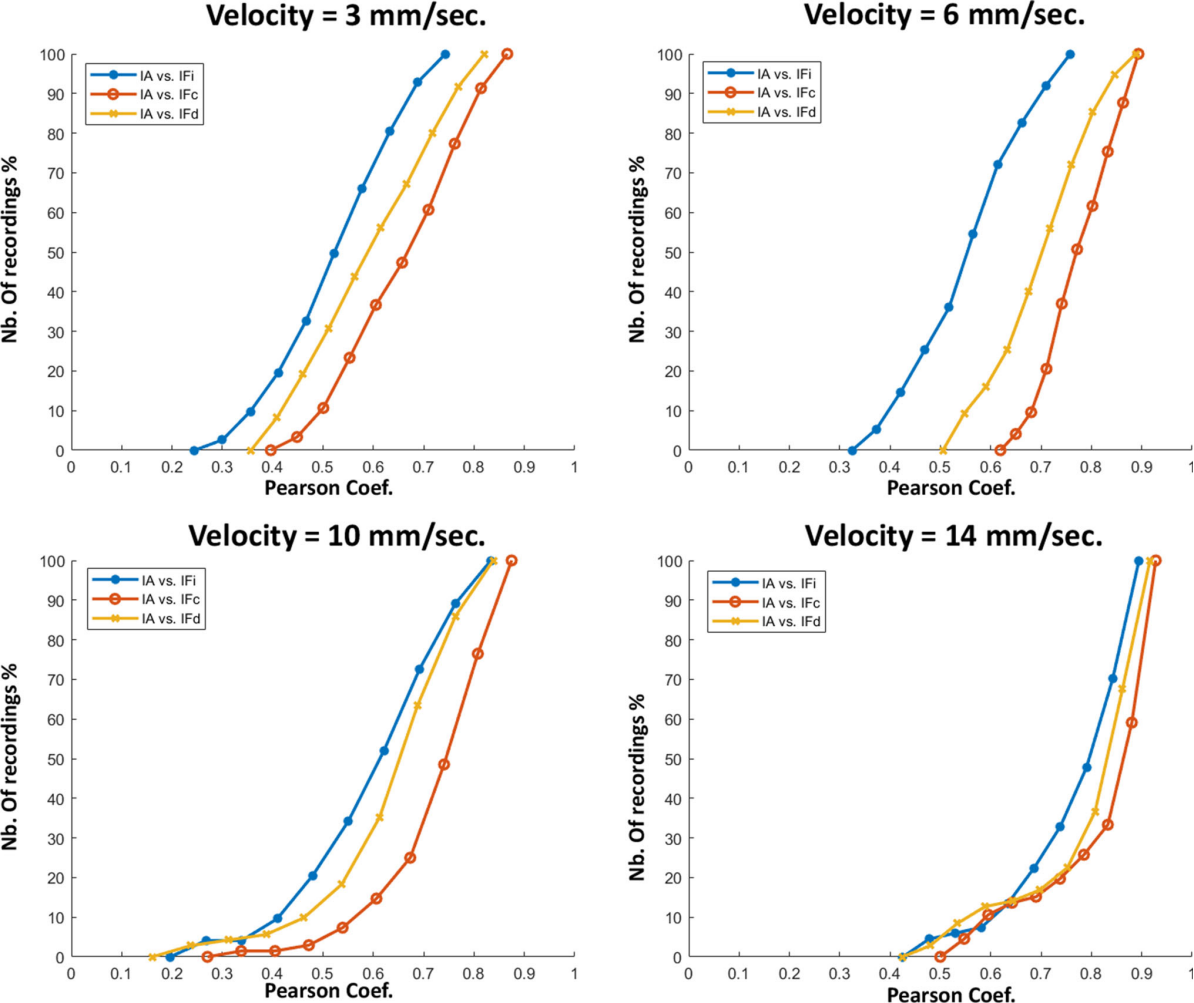
Cumulative histograms of the Pearson coefficients (in terms of percentage of nb. of recordings) for all comparisons between the force indicators and audio indicator, and for the four tested velocities

It is also possible to analyze from the 6th column of the table that the obtained results seem to be not so dependent on the signal processing parameters: the mean and standard deviation of the Pearson coefficients during the optimization procedure are still high and involving low variability, demonstrating the robustness of the presented comparison approach.

Concerning the optimized parameters, we can observe that the best average Pearson coefficients are obtained with $lpf_a$ and $lpf_f$ values set on 1. This means that the related information between force and audio is located mainly in the low-frequency dynamics.

Figure 11 shows the histograms of the Pearson coefficients between the three force indicators and the audio indicator for the four tested insertion velocities. The histograms confirm the results shown in Table 2: for the three insertion velocities, the curvature is the force indicator that better correlates with the audio indicator. It is also possible to see that the derivative force indicator behaves better than the local intensity indicator for all the tested velocities. It is essential to notice that, in general, the fact that audio dynamics are related to force dynamics does not depend on the insertion velocity. If we analyze, for example, curvature and derivative, it is possible to see that most of the recordings, regardless of the insertion velocity, present middle and high Pearson coefficient values.

The analysis made in Fig. 11 is confirmed by the cumulative histograms displayed in Fig. 12, where it is possible to observe that for the four tested velocities, more than $70\%$ of the correlations between the force curvature indicator $IF_c$ and the audio indicator involve coefficients higher than 0.6. Concerning the derivative force indicator $IF_d$, more than $60\%$ of the recordings, regardless of the insertion velocity, present coefficient values higher than 0.6 when correlated with the audio indicator. Although the force intensity indicator $IF_i$ seems to have low values compared to the other two indicators, the coefficient values of more than $50\%$ of the recordings are over 0.5, which can be considered high if we consider the completely different nature between audio and force sensors.

**Fig. 13:**
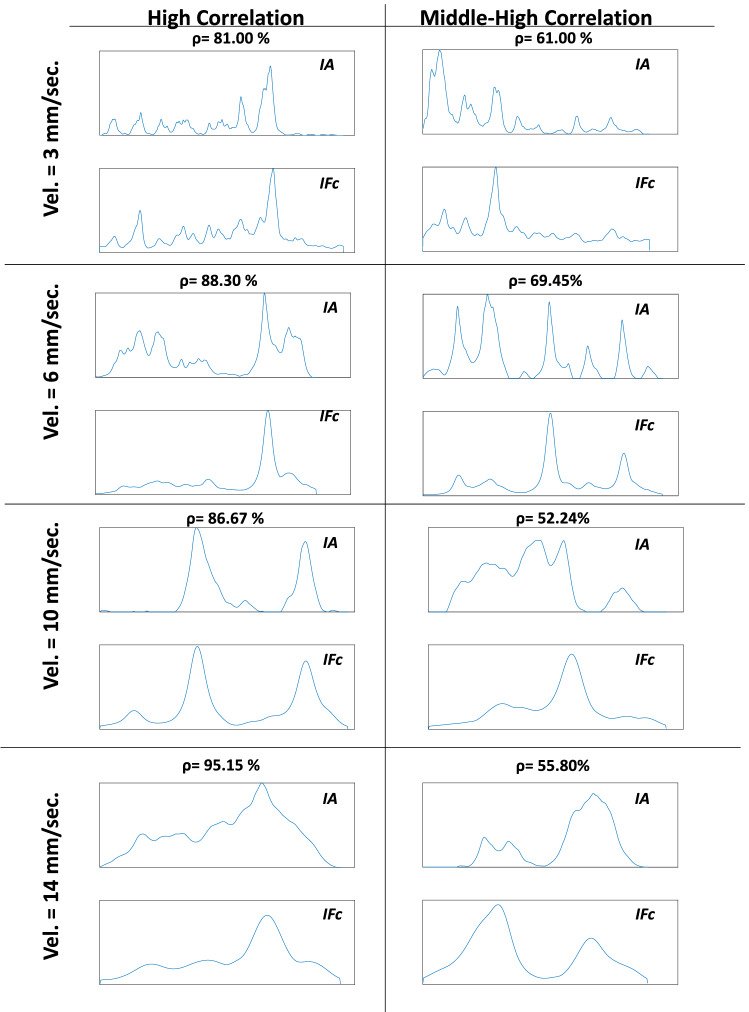
Examples of force curvature and audio indicators where high and middle-high correlations were obtained

Figure 13 gives us an idea of how the force curvature indicator and the audio indicator, which present high or middle-high Pearson coefficient value (displayed in percentage in the figure), looks like. The figure shows eight recordings taken from the implemented database; two recorded per tested insertion velocity. Examples of high ($\rho >0.8$) and middle-high ($0.5<\rho <0.7$) Pearson coefficient are displayed. It is possible to observe that audio and force low-frequency dynamics are strongly related, showing how part of the force’s information could also be extracted from the audio.

It is important to point out in Fig. 13 that the higher the velocity, the smoother is the force signal. This is because the force change with the needle insertion speed. When the needle insertion velocity increases, the force decreases [8]. When the velocity is low, tiny tissue breakage of internal structures results in peaks in the force signal. When the velocity is higher, there is no reaction of force for these structures, and it is possible only to identify reactions in the force resulting from significant punctures. This property can also explain what happens in Fig. 11 where the higher the insertion velocity, the higher the percentage of recordings having high correlation values between audio and force indicator since only the main puncture events are assessed.

**Fig. 14:**
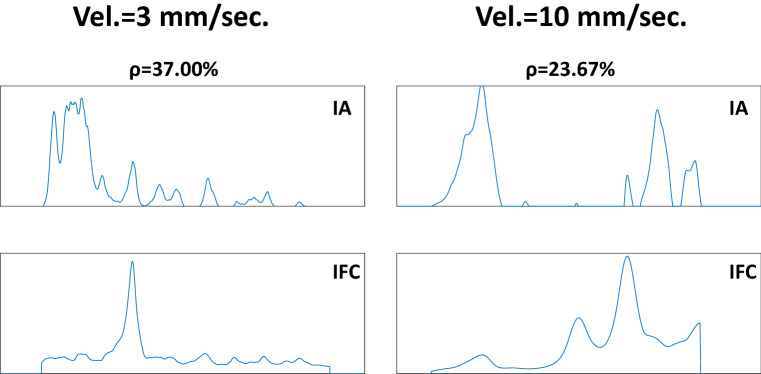
Examples of force curvature and audio indicators where low correlations were obtained

Although a high percentage of recordings present correlations between force and audio indicators over 0.5, there are cases (see Fig. 11) where low correlations are obtained. Figure 14 displays two examples of low correlations ($\rho <0.4$) for velocities of 3 mm/s and 10 mm/s. These low correlations may have various reasons. First, a force signal peak can sometimes occur with no tissue rupture, which is always necessary to create an audio excitation. Another possible reason could be tiny errors during the synchronization process between the audio and the force signals.

Additionally, it should be noted that recordings displaying low correlation in the histogram of Fig. 11 are not necessarily the same for each force indicator. A correlation between audio and two different force indicators can result in different correlation coefficients. This can occur because different force indicators can enhance different characteristics of the force signal that may or may not be correlated with the audio envelope.

### Event-to-event correlation

**Fig. 15:**
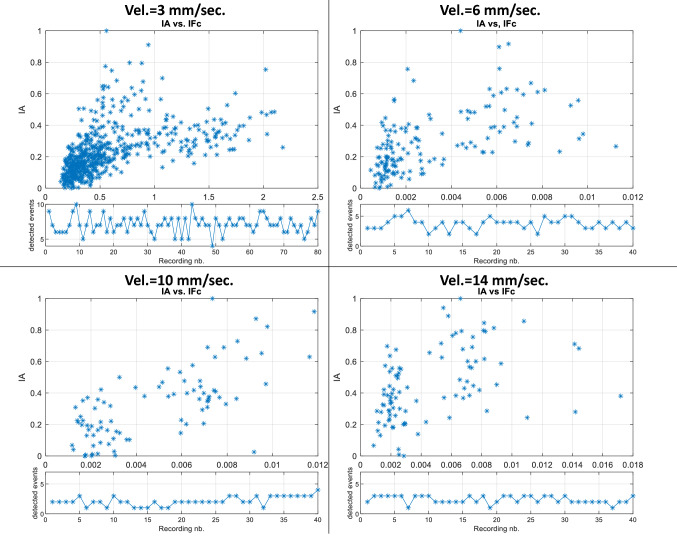
Event-to-event correlation between the maximal values of the audio and force indicators inside the detected events

Figure 15 shows the scatter plots of the maximal values of the detected events for force and audio indicators (as explained in Sect. 3.1.3) for the best force indicator $IF_c$. It is possible to see a clear linear trend between the event’s maximal values in force and audio for the four tested velocities. Higher is the curvature of the force during the puncture; higher is the probability that the audio excitation will excite with higher energy.

Table 3 displays the confusion matrix of the identifiable events in the curvature force indicator and the corresponding identifiable event in the audio indicator. True positives (TP) are the events that are identifiable in $IF_c$ and that have a corresponding identifiable event in *IA*. False negatives (FN) are the events identifiable in $IF_c$ that does not produce a significant excitation in *IA*. The TP values for the four tested velocities are over $78\%$, with even $100\%$ of force events that produce an audio excitation for the insertion velocity of 6 [mm/s], confirming that most of the puncture events visible in force are also visible in audio.

## Discussion and conclusion

In this work, we investigated if audio signals recorded from the proximal end of the needle during needle insertion can provide important information about the occurring events during the needle insertion process. For that, we explored the audio dynamics generated from the tip/tissue interaction by comparing them to dynamics measured with a force sensor used as reference.

Different needle insertion velocities were tested to analyze the signal dynamical responses in both sensors. The main idea was to observe the effect of different characteristics of force on the audio excitation to evaluate the relationship between the sensors’ responses.

**Table 3:** Confusion matrix of detected events in the curvature force indicator and in the audio indicator for all insertion velocities, showing the true positives (TP), the false negatives (FN) and the number of identified events

Insertion velocity	3 mm/sec.	6 mm/sec.	10 mm/sec	14 mm/sec.
TP	193 (87.7%)	62 (100%)	52 (78.8%)	44 (88%)
FN	27 (12.3%)	0 (0%)	14 (21.2%)	6 (12%)
Total nb. of events	220	62	66	50

The results confirm what has been presented in [9]. Audio contains valuable information for monitoring needle tip/tissue interaction dynamics. Significant dynamics that can be obtained from a well-known sensor as force can also be extracted from audio. The results also show that the audio guidance approach is robust to insertion velocities since the signal-to-signal and event-to-event correlation performances do not change between the four tested velocities.

The correlation analysis demonstrates that audio and force characteristics, even resulting from sensors of an entirely different nature, can be strongly related, confirming the wealth of information that an audio signal can contain.

The idea of this work was not to derive force from audio but to use it as the indicator of events that should be identified in the audio signal. Knowing and recognizing these important events in the audio signals proves the presented approach’s usability for needle guidance.

Extracting force from audio could be extremely difficult given the complex relationship between both modalities. However, using a large puncture database and machine learning algorithms would be possible to obtain a transfer function that could translate audio into force. This is a path that could be explored in the future, but first, it should be studied if, in terms of guidance information, the surgeon needs to know the exerted force or if it is more important to have access to the main events that can occur during the needle insertion (punctures or tissue-tissue passage for example). Although the signal processing strategy proposed in this work was performed for offline signal analysis, audio could provide these events in real time: the linear filter and homomorphic envelope used in this work for audio processing could be easily adapted to operate in real time.

Even though this work does not intend to replace force with audio, it is important to mention that force has been mainly used for research studies of needle puncture into soft tissue, but, up to our knowledge, it has not been adopted for regular clinical use. This may be due to the difficult integration of the force sensing module to the tool. Audio sensing is a low-cost technology that can be easily adapted to a tool: a tissue rupture will always result in an audio wave that will propagate through the shaft and that can be picked up at the proximal end of the tool. The location of the sensor does not limit audio excitations resulting, for example, from needle punctures. Additionally, audio involves fast dynamical changes (in the order of milliseconds) when a change in the process occurs, while the time constants involved in the dynamical changes in force sensing are slow. This can be important for robotic applications.

As future steps, further studies are needed to understand the audio signal dynamics that result in low correlations with the force indicators. Also, the operation of this audio concept in a real clinical scenario (noisy environment, variability between users) should be further tested and validated.
